# Selecting Initial Conditions for Trajectory-Based Nonadiabatic Simulations

**DOI:** 10.1021/acs.accounts.4c00687

**Published:** 2025-01-09

**Authors:** Jiří Janoš, Petr Slavíček, Basile F. E. Curchod

**Affiliations:** † Department of Physical Chemistry, University of Chemistry and Technology, Technická 5, Prague 6, 166 28, Czech Republic; ‡ Centre for Computational Chemistry, School of Chemistry, University of Bristol, Bristol BS8 1TS, United Kingdom

## Abstract

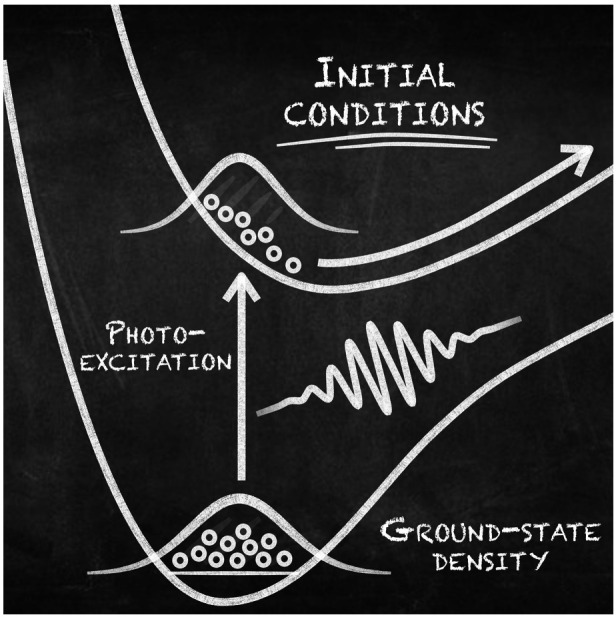

Photochemical reactions have always been the source of a great deal of mystery. While classified as a type of chemical reaction, no doubts are allowed that the general tenets of ground-state chemistry do not directly apply to photochemical reactions. For a typical chemical reaction, understanding the critical points of the ground-state potential (free) energy surface and embedding them in a thermodynamics framework is often enough to infer reaction yields or characteristic time scales. A general working principle is that the energy profile along the minimum energy paths provides the key information to characterize the reaction. These well-developed concepts, unfortunately, rarely stretch to processes involving the formation of a nonstationary state for a molecular system after light absorption.

Upon photoexcitation, a molecule is likely to undergo internal conversion processes, that is, changes of electronic states mediated by couplings between nuclear and electronic motion, precisely what the celebrated Born–Oppenheimer approximation neglects. These coupled electron–nuclear processes, coined *nonadiabatic processes*, allow for the molecule to decay from one electronic state to the other nonradiatively. Understanding the intricate nonadiabatic dynamics is pivotal to rationalizing and predicting the outcome of a molecular photoexcitation and providing insights for experiments conducted, for example, in advanced light sources such as free-electron lasers.

Nowadays, most simulations in nonadiabatic molecular dynamics are based on approximations that invoke a near-classical depiction of the nuclei. This reliance is due to practical constraints, and the classical equations of motion for the nuclei must be supplemented by techniques such as surface hopping to account for nonadiabatic transitions between electronic states. A critical but often overlooked aspect of these simulations is the selection of initial conditions, specifically the choice of initial nuclear positions and momenta for the nonadiabatic dynamics, which can significantly influence how well the simulations mimic real quantum systems across various experimental scenarios. The conventional approach for generating initial conditions for nonadiabatic dynamics typically maps the initial state onto a nuclear phase space using a Wigner quasiprobability function within a harmonic approximation, followed by a second approximation where the molecule undergoes a sudden excitation.

In this Account, we aim to warn the experienced or potential user of nonadiabatic molecular dynamics about the possible limitations of this strategy for initial-condition generation and its inability to accurately describe the photoexcitation of a molecule. More specifically, we argue that the initial phase-space distribution can be more accurately represented through molecular dynamics simulations by using a quantum thermostat. This method offers a robust framework that can be applied to large, flexible, or even solvated molecular systems. Furthermore, the reliability of this strategy can be benchmarked against more rigorous approaches such as path integral molecular dynamics. Additionally, the commonly used sudden approximation, which assumes a vertical and sudden excitation of a molecule, rarely reflects the excitation triggered by laser pulses used in actual photochemical and spectroscopic experiments. We discuss here a more general approach that can generate initial conditions for any type of laser pulse. We also discuss strategies to tackle excitation triggered by a continuous-wave laser.

## Key References

- SuchanJ.; HollasD.; CurchodB. F. E.; SlavíčekP.On the Importance of Initial Conditions for Excited-State Dynamics. Faraday Discuss.2018, 212, 307–33030259011 10.1039/c8fd00088c.^1^*This work discusses in detail the photoexcitation process resulting from different light sources–laser pulses, CW field–and the impact on nonadiabatic molecular dynamics.*
- PrljA.; MarsiliE.; HuttonL.; HollasD.; ShchepanovskaD.; GlowackiD. R.; SlavíčekP.; CurchodB. F. E.Calculating Photoabsorption Cross-Sections for Atmospheric Volatile Organic Compounds. ACS Earth and Space Chemistry2022, 6, 207–21735087992 10.1021/acsearthspacechem.1c00355PMC8785186.^2^*This work investigates different approximations for the ground-state nuclear probability distribution of (flexible) atmospheric molecules in the context of photoabsorption.*
- PrljA.; HollasD.; CurchodB. F. E.Deciphering the Influence of Ground-State Distributions on the Calculation of Photolysis Observables. J. Phys. Chem. A2023, 127, 7400–740937556330 10.1021/acs.jpca.3c02333PMC10493954.^3^*This work highlights the artifacts created by the harmonic Wigner sampling when employed for molecules with low-frequency modes that are photoactive.*
- JanošJ.; SlavíčekP.; CurchodB. F. E.Including photoexcitation explicitly in trajectory-based nonadiabatic dynamics at no cost. J. Phys. Chem. Lett.2024, 15, 10614–1062239405399 10.1021/acs.jpclett.4c02549PMC11514012.^4^*This work introduces the promoted density approach (PDA) for the implicit inclusion of a laser-pulse photoexcitation in nonadiabatic molecular dynamics at the level of the initial conditions.*

## 1 Introduction

Simulating the photochemistry of a molecule often requires the description of the subtle coupling between electronic and nuclear motion, the so-called *nonadiabatic effects* that are neglected within the Born–Oppenheimer approximation. Nonadiabatic molecular dynamics aims to describe the dynamics of molecules beyond the Born–Oppenheimer picture and has mostly focused on the dynamics of nuclear wavepackets. The initial nuclear wavepacket, representing the molecular state of the system following photoexcitation, is nonstationary and, as such, will exhibit a nontrivial time evolution. If the nuclear wavepacket reaches a region where electronic states get close in energy, the coupling between nuclear and electronic motion will result in a transfer of (part of) the nuclear wavepacket to a different electronic state. These nuclear wavepackets can explore different regions of configurations space, some prone to nonadiabatic transitions. The determination of experimental observables characterizing the photochemistry of molecules, therefore, relies on a proper simulation of this out-of-equilibrium nonadiabatic dynamics.

The past 30 years have witnessed the development of a broad range of techniques to simulate the nonadiabatic dynamics of molecules.^5,6^ Some methods are deeply rooted in a numerically exact solution of the time-dependent molecular Schrödinger equation, benefiting from high accuracy but often for a reduced number of nuclear degrees of freedom.^7^ An alternative approach represents the nuclear wave functions in a basis of *coupled* multidimensional traveling Gaussian functions, also called trajectory basis functions (TBFs).^8−10^

Yet, the favored strategy to mimic the dynamics of nuclear wavepackets in their full dimensionality is to use a swarm of classical trajectories. These trajectories can be mutually coupled to incorporate some nuclear quantum effects^11^ or independent to simplify the formalism while offering a first approximation to the nonadiabatic molecular dynamics.^12^ Trajectory surface hopping (TSH), proposed in its most widely used “fewest-switches” flavor in 1990,^13^ is the most famous member of this family of methods. In TSH, a trajectory is initiated in a given excited electronic state of interest and propagated classically together with a proxy measuring the strength of nonadiabatic effects. This measure of nonadiabaticity can trigger a stochastic hop to a different electronic state, meaning that the classical trajectory would, from this time, be propagated according to the nuclear forces dictated by this specific electronic state. Multiple independent trajectories are required to obtain an approximate depiction of the nuclear wavepacket dynamics.

Nonadiabatic molecular dynamics simulations can provide a plethora of details about the mechanisms underlying the nonradiative decay of a molecule following its photoexcitation. A major effort in the field is also currently ongoing to use the results of nonadiabatic dynamics simulations to calculate actual experimental observables,^14−18^ offering a more direct and consistent connection to (time-resolved) experiments, in particular those conducted at advance light sources like free-electron lasers.^19,20^

**Figure 1:**
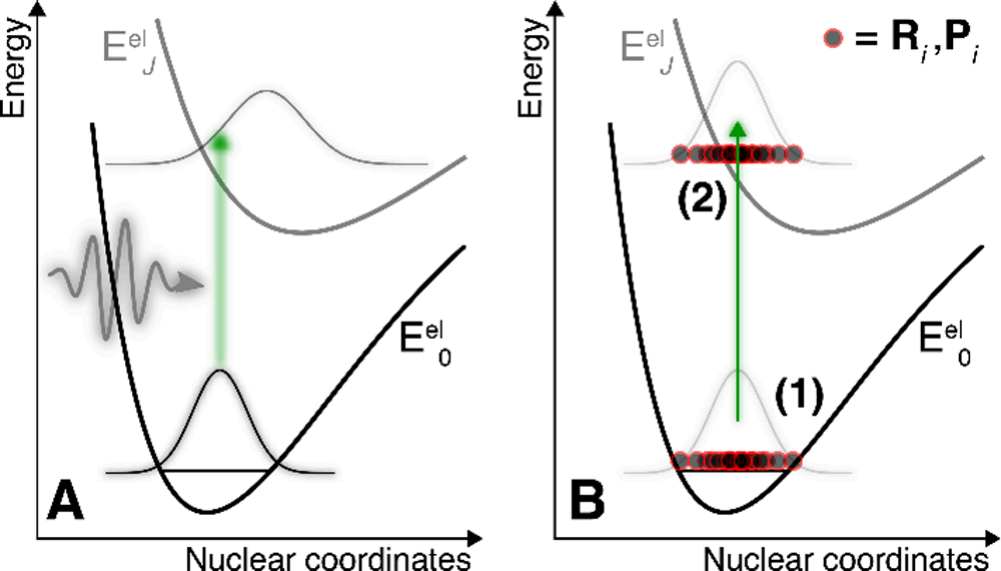
Photoexcitation process in nonadiabatic dynamics. (A) A quantum-dynamics perspective. The nuclear wave function of a molecule in its ground electronic (*E*_0_^el^) and vibrational state transfers its amplitude to an excited electronic state *J* under the influence of a short laser pulse. (B) An approximate trajectory-based perspective. Step (1): a set of *N*_IC_ initial nuclear positions and momenta (**R**_*i*_ and **P**_*i*_, red-black circles) are sampled from an *approximate* probability distribution. Step (2): these nuclear positions and momenta are promoted vertically, within the *sudden* excitation approximation (by a δ-pulse), to a given electronic state *J*, representing the formation of a perfect nuclear wavepacket in state *J*.

Surprisingly, despite the decades of advances in nonadiabatic molecular dynamics methods described above, the very first step of a photochemical reaction, the photoexcitation (see Figure 1A), is often described at an intuitive level. Given the impact that photoexcitation can have on a photochemical process, its rather poor description in current simulations is quite worrying. Within trajectory-based nonadiabatic simulations, the details of the photoexcitation can only be controlled by an adequate choice of initial conditions, which are meant to encode the photoexcitation process. The term “initial conditions” is, however, rather ill-defined, and we propose here to use the following descriptor for the set of *N*_IC_ initial conditions: , with **R**_*i*_ and **P**_*i*_ being positions and momenta of all nuclei for the initial condition *i* and *J*_*i*_ being the label of the (excited) electronic state where the trajectory *i* is started at time *t*_*i*_.

The process of determining initial conditions in nonadiabatic dynamics is often split into two steps: (1) obtain an approximate ground-state nuclear distribution for the molecule (obtain **R**_*i*_ and **P**_*i*_) and (2) transfer this distribution into the excited electronic state(s) of interest (determine *J* and *t*), see Figure 1B. The rationale for projecting the ground-state distribution to an excited electronic state (Figure 1B) follows from first-order time-dependent perturbation theory,^21^ which shows that the photoexcitation of a two-state molecular system using an infinitely short laser pulse (a δ-pulse) leads to the projection of the initial ground-state nuclear wave function on the targeted excited electronic state.^a^ The molecular state produced with such a protocol is *only* representative of a photoexcitation with a δ-pulse leading to a perfect projection of the initial nuclear wave function (or, at least, with a laser pulse with an energy bandwidth broad enough to encompass all the vibrational states, in the excited electronic state, required to perfectly project the initial ground-state nuclear wave function).^b^ This approximation is often referred to as the “sudden excitation” and implies that *t*_*i*_ = 0 for all initial conditions. Any laser pulse deviating from this idealized δ-pulse would change the initial nuclear wavepacket.^22^ The approximate nature of initial conditions in nonadiabatic molecular dynamics does not stop here, and even step 1, the sampling of an initial ground-state distribution, often relies on rather strong approximations.

This Account aims to (i) sound the alarm on the potential consequences of an improper choice of initial conditions in trajectory-based nonadiabatic molecular dynamics simulations and (ii) spotlight recently developed strategies to sample the ground-state probability distribution of a molecule and describe its photoexcitation adequately, in the gas phase and beyond.

## 2 Sampling an Approximate Ground-State Nuclear Probability Distribution: Beyond the Harmonic Wigner Strategy

In this section, we discuss different techniques to sample approximately the ground-state nuclear distribution originating either from a single (stationary) vibrational wave function χ_0_(**R**) (step 1 in Figure 1) or, more generally, from an ensemble of vibrational wave functions, e.g., at thermal equilibrium. What we mean by “sampling” in trajectory-based methods for nonadiabatic molecular dynamics is the generation of a set of initial nuclear positions **R**_*i*_ and initial nuclear momenta **P**_*i*_ (parts of our initial-condition descriptor ) from a distribution of the possible nuclear momenta and positions for the system. This set of initial conditions should provide the best phase-space representation of the underlying quantum system, keeping in mind that we intend to use (classical) trajectories to depict molecular quantum dynamics.

In the following, we discuss the most commonly employed strategies to sample initial conditions based on a Wigner distribution obtained from a harmonic approximation for the ground state potential energy surface. We then advocate for an alternative approach to recover a nuclear phase-space distribution based on *ab initio* molecular dynamics with a so-called quantum thermostat.

### 2.1 Harmonic Wigner Sampling

The Wigner distribution transforms the nuclear wave function into a quasiprobability phase-space distribution,^23^
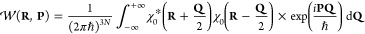
1The term “quasiprobability” is used here due to the possible presence of negative values in the Wigner distribution (see below). Besides this, the Wigner distribution for a nuclear wave function behaves as if it were a probability distribution for both the nuclear coordinates and the nuclear momenta since its marginal probability distributions correspond to the exact probability distributions of coordinates and momenta, i.e.,  and . Thus, one could sample from  a set of **R**_*i*_ and **P**_*i*_ to serve as initial conditions for trajectory-based methods.

**Figure 2:**
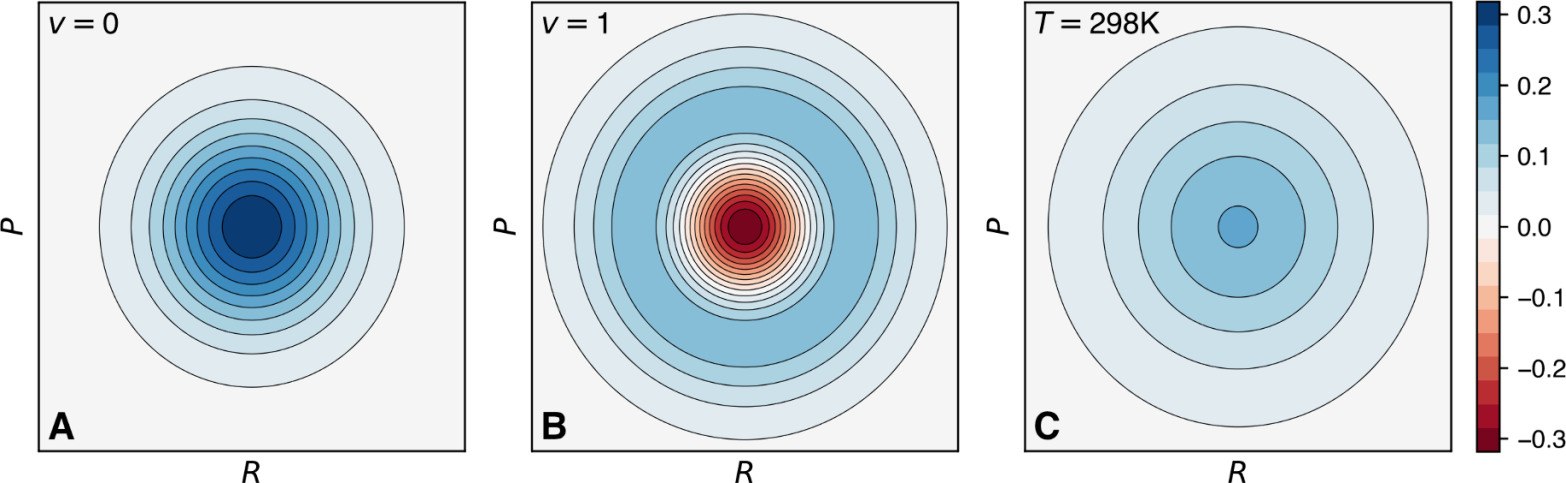
Wigner distribution for a harmonic oscillator (ω = 0.001 au and *m* = 32 amu) in (A) vibrational state *v* = 0 and (B) vibrational state *v* = 1 (note the presence of a negative region in the Wigner distribution). (C) Thermal Wigner distribution for the same harmonic oscillator but at *T* = 298 K (note the absence of negative regions in the distribution).

In the most practical use of the Wigner distribution in nonadiabatic molecular dynamics, one invokes a harmonic approximation for the ground-state potential energy surface near its equilibrium geometry. The nuclei of the molecule are represented here via their normal modes as a combination of (uncoupled) quantum harmonic oscillators.^1,12,24^^,^^c^ Under this harmonic approximation, which only requires a ground-state optimized geometry for the molecule of interest and the corresponding harmonic vibrational frequencies, the Wigner distribution of a single normal mode equals^25^
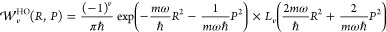
2where *L*_*v*_ are the Laguerre polynomials (and we note that the distribution is centered at zero for positions and momenta as it uses a normal mode representation). Taking the ground vibrational state (*v* = 0), the Wigner distribution simplifies to
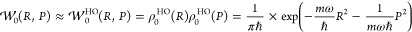
3with ρ_0_^HO^(*R*) and ρ_0_^HO^(*P*) being the ground-state harmonic nuclear densities in position and momentum space, respectively. A one-dimensional depiction of  for the first two lowest vibrational states is given in Figure 2, highlighting the negative region of the distribution for *v* = 1 (Figure 2B). The harmonic Wigner distribution for all normal modes, , is then constructed by multiplying the single-mode Wigner distributions.

For a one-dimensional harmonic oscillator in thermal equilibrium, the Wigner distribution reads^21^
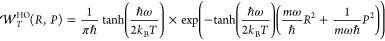
4The thermal harmonic Wigner distribution is always positive-definite (Figure 2C), allowing the sampling of nuclear positions (**R**_*i*_) and momenta (**P**_*i*_) for molecular systems in thermal equilibrium, something that the harmonic Wigner distribution and its associated negative values for excited vibrational states would not permit trivially. For additional details on the use of harmonic Wigner distributions in nonadiabatic dynamics, the reader is referred to refs (1, 12, and 24).

The harmonic Wigner distribution produces an approximate ground-state nuclear distribution for general molecules efficiently and is therefore routinely used in the field of nonadiabatic dynamics. The generated initial conditions will, on average, display a classical total energy that matches the average quantum energy, i.e., the zero-point energy (ZPE). Given its simplicity, sampling of initial conditions with a harmonic Wigner strategy is implemented in most packages for nonadiabatic molecular dynamics. The sampled nuclear positions can also be used within the nuclear ensemble approach (NEA) to calculate the photoabsorption cross-sections. The NEA, which is a numerical realization of the reflection principle,^26^ uses vertical excitation energies and transition dipole moments calculated on each sampled geometry to build a photoabsorption cross-section (for additional information on the NEA, the reader is referred to refs (2, 12, 27, and 28)).

However, great care is required when using the harmonic Wigner distribution for molecules exhibiting low-frequency vibrational modes, including the important case of solvated chromophores. Given that the common implementations of the harmonic Wigner approximation employ normal modes described in rectilinear coordinates (from electronic-structure packages), a danger exists that distortion along (rectilinear) low-frequency modes may couple with high-energy modes and results in artificially high-energy initial molecular structures.^3^ For example, the torsion around a methyl group, in the rectilinear coordinate, can artificially induce a stretching of the C–H bonds, that is, a coupling with C–H stretches.^29^ A proposed strategy to alleviate this artifact is to remove the contribution of low-frequency modes (typically under 500 cm^–1^) from the sampling,^2,24,30−33^ but care is needed with this approach as shown below. While this type of artificial coupling may not significantly alter the outcome of nonadiabatic dynamics when the modes impacted are not playing a role in the photochemistry of the system, recent examples have shown the dramatic effects that this breakdown of the harmonic Wigner sampling can generate (see the example of methyl hydroperoxide discussed below).

### 2.2 Sampling with Molecular Dynamics: From Classical to Quantum Thermostats

*Ab initio* Born–Oppenheimer molecular dynamics (BOMD) is a possible alternative to the harmonic Wigner distribution for generating a ground-state nuclear probability distribution by running a long trajectory in the ground electronic state of the molecule of interest. BOMD becomes especially convenient when initial conditions are needed for a molecular system of interest in an environment. Typically, the BOMD sampling is performed with a thermostat aiming at modeling a classical system at a certain temperature. As BOMD represents the nuclear degrees of freedom in a purely classical way, a sampling emanating from a BOMD trajectory at a temperature of 300 K provides molecular geometries with a much narrower distribution of bond distances and angles than the one representative of a distribution at the ZPE.^34^ A distribution closer to that of ZPE can be obtained by artificially increasing the temperature of the BOMD simulation, but this technique should be considered at best as a patch as it ignores the very different distribution of energy for oscillators having different frequencies.

A practical and more rigorous strategy to produce an approximate ground-state phase-space distribution using BOMD is to couple it with a quantum thermostat (QT).^1^ The QT is based on the idea that both the classical and quantum distributions for harmonic systems are Gaussian; the quantum system can thus be mapped on a classical system at a (frequency-dependent) elevated temperature. The QT can be designed within the formalism of the generalized Langevin equation (GLE), where the thermostat guarantees to keep the normal modes of a molecule at different frequency-dependent “temperatures”, reproducing rigorously the phase-space distributions corresponding to quantum harmonic oscillators.^35−37^ In other words, the QT is a thermostat ensuring that each normal mode of a molecule reaches its ZPE from a harmonic perspective, while the underlying dynamics used for the sampling do not rely on the harmonic approximation. QT-BOMD simulations are formally exact for a system of uncoupled harmonic oscillators but perform well for (moderately) anharmonic molecules with low-frequency modes or to sample high-frequency modes, where quantum effects may dominate.^1,2,38,39^ While the workflow associated with the QT-BOMD is more computationally demanding than that of the harmonic Wigner sampling, it is of a similar cost to BOMD.

Perhaps more importantly, the QT approach combined with the BOMD permits the retrieval of approximate (nuclear) *phase-space* distributions. This contrasts with other methods that only provide an in principle exact nuclear *position* distribution, such as the path-integral molecular dynamics approach (PIMD, exact distribution at finite temperature *T*) or the diffusion quantum Monte Carlo method (exact nuclear ground-state wave function). The PIMD sampling strategy ignores the momentum part of the phase-space distribution or approximates it with a classical Boltzmann distribution. Sampling only the nuclear position space is still valuable for the calculation of photoabsorption cross-sections or as initial conditions for photochemical reactions with a highly sloped excited potential energy surface in the Franck–Condon region. The PIMD-based sampling was thus successfully used on several occasions.^40,41^ As the QT formalism has an unknown error brought about by the design of the GLE thermostats for the harmonic cases, one can also use the position distribution provided by a PIMD simulation to validate the nuclear phase-space distribution obtained with QT-BOMD (see below for an example).

### 2.3 A Parenthesis on Zero-Point Energy Leakage

Using a Wigner sampling to describe the initial state of a molecule (for example, prior to photoexcitation) accurately maps the initial vibrational state, including the zero-point energy (ZPE) in each mode. In quantum systems, high-frequency modes retain more energy than low-frequency modes and each mode will retain at least its zero-point energy. In contrast, classical systems assume an equal energy distribution across all modes, with no minimal zero-point energy limits. As a result, a classical evolution (based on an initial quantum distribution of the initial vibrational state) tends to cause an unnatural energy flow from high-frequency modes to low-frequency modes, defining ZPE leakage. ZPE leakage can affect the results of nonadiabatic molecular dynamics methods based on a swarm of classical trajectories (TSH) when long time scale dynamics are considered,^24,42^ and different corrections for trajectory-based techniques were suggested.^42−44^

### 2.4 Photolysis of Methyl Hydroperoxide as an Example

**Figure 3:**
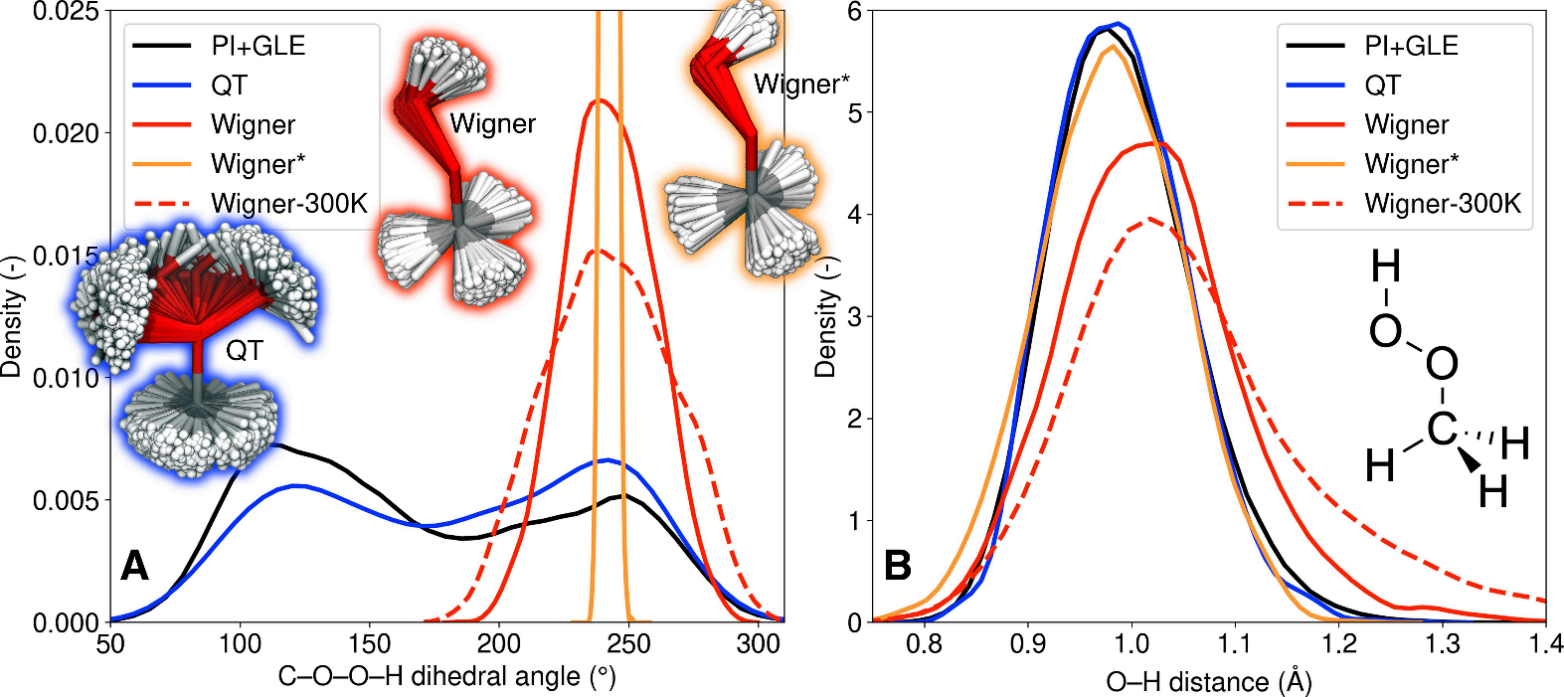
Ground-state density distribution for (A) the C–O–O–H dihedral angle and (B) the O–H bond of MHP obtained from different sampling procedures, namely Wigner, Wigner* (lowest-frequency mode removed in sampling), QT-BOMD, PI+GLE, and Wigner at 300 K (Wigner-300 K). Reproduced from ref (3). Available under a CC-BY license. Copyright 2023 A. Prlj, D. Hollas, B. F. E. Curchod.

Let us now illustrate the performance of the strategies introduced previously for a molecular example exhibiting a low-frequency vibrational mode, namely, methyl hydroperoxide (MHP, H_3_COOH). MHP is one of the most abundant organic peroxides in our atmosphere,^45^ with different wavelength-dependent photolysis channels leading to the release of OH, H, or even O. MHP possesses a low-frequency normal mode at 201 cm^–1^ (MP2/aug-cc-pVDZ) connected to the C–O–O–H torsional mode (see molecular structure in the inset of Figure 3). This low-energy mode was recently shown to cause issues for the sampling of initial conditions using the conventional harmonic Wigner distribution.^2,3^ First of all, the presence of the low-energy mode means that the harmonic Wigner distribution does not properly describe the broad distribution of the C–O–O–H dihedral angle (Figure 3A). Changing the “temperature” of the harmonic Wigner sampling does not improve the result. Using QT-BOMD leads to an improved description of the distribution for the C–O–O–H dihedral angle, in agreement with the path-integral result (PI+GLE) (we note that nuclear momenta were also compared in the SI of ref (3)). But the most critical issue with the harmonic Wigner sampling, in this case, is caused by the spurious coupling between the low-frequency C–O–O–H torsional mode and the high-frequency O–H stretch of MHP that artificially prolongs the O–H bonds (see Figure 3B). Removing the low-frequency mode from the sampling prevents the artificial O–H stretch (“Wigner*”, Figure 3B) and provides distributions in agreement with QT-BOMD and PI+GLE, at the cost, however, of a very narrow distribution for the C–O–O–H dihedral angle (Figure 3A).

**Figure 4:**
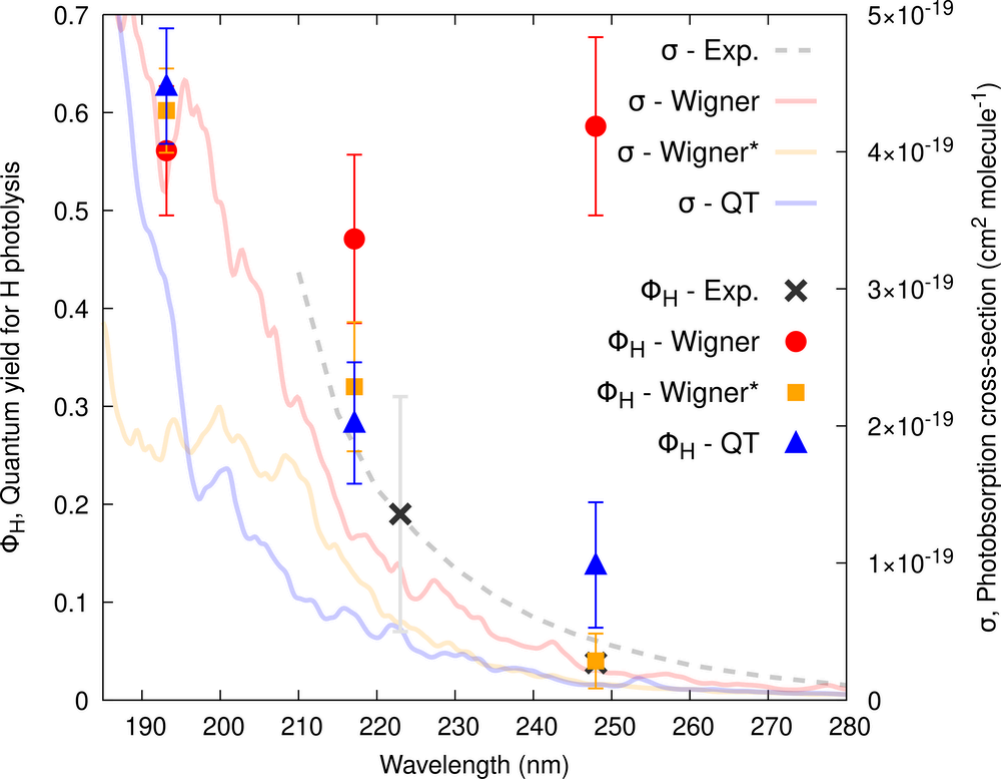
Calculated photoabsorption cross-sections (σ, curves) and wavelength-dependent quantum yields (ϕ_H_, symbols) for the H atom photolysis from MHP. σ and ϕ_H_ were obtained from the NEA and TSH dynamics, respectively, based on different sampling procedures: Wigner, Wigner*, and QT-BOMD. The dashed curve and black crosses correspond to experimental data.^49,50^ Reproduced from ref (3). Available under a CC-BY license. Copyright 2023 A. Prlj, D. Hollas, B. F. E. Curchod.

What is the impact of these artifacts on the prediction of photoabsorption and photochemical properties? While the trends described above may seem unimportant at first glance, the issue is that one of the excited electronic states of MHP exhibits n(OO)σ*(OH) character. Hence, the geometries sampled from the harmonic Wigner sampling with a stretched O–H bond exhibit low-energy transitions toward the n(OO)σ*(OH). The nonadiabatic molecular dynamics (TSH) initiated from these initial conditions leads to an artificially large number of H photolysis events at low energy (∼59% for Wigner vs 4% in the experiment at 248 nm, Figure 4). The initial conditions obtained with QT-BOMD or Wigner* lead to a better agreement with the experiment. However, the photoabsorption cross-section obtained with Wigner* (orange curve in Figure 4) lacks intensity at short wavelength due to the narrow distribution of the C–O–O–H dihedral angle. Hence, removing low-energy frequencies from the sampling should be done with care if the modes are possibly photoactive (from a photoabsorption or photochemical perspective). Thus, QT-BOMD appears as the most reliable strategy for sampling the ground-state nuclear phase-space distribution of molecular systems and as such has already been used in several works successfully (see for example refs (1, 2, 46−48)). We should stress, however, that even the best possible sampling of the ground-state distribution, e.g., based on an exact Wigner distribution, would not solve the inherent problems of using a swarm of classical trajectories in nonadiabatic dynamics, such as the ZPE leakage mentioned above.

## 3 Photoexcitation: Beyond the Sudden-Excitation Approximation

Considering at this point that we can obtain an adequate approximation to the ground-state nuclear phase-space distribution for our molecular system, we can move to step (2) depicted in Figure 1, the photoexcitation process. In the following, we discuss different strategies to treat the photoexcitation step beyond the crude sudden-excitation approximation introduced in Section 1. This section does not discuss approaches that include *explicitly* a laser pulse (or other time-dependent electric fields) in the simulation, since they are typically too computationally expensive for practical applications and often inaccurate for anything but laser pulses with a few cycles (especially for methods like TSH^51,52^). The reader interested in these techniques can consult refs (53−58).

### 3.1 Including a Laser Pulse Implicitly via the Initial Conditions

A rather common approach to approximately account for the effect of a laser pulse in a nonadiabatic molecular dynamics simulation is to perform *energy windowing* of the initial conditions. The central idea is to calculate a photoabsorption cross-section (using the NEA, for example) based on the nuclear positions sampled from the approximate ground-state distribution and superimpose to these cross-sections an energy window around the central pulse frequency, with a width proportional to the pulse bandwidth.^59^ Each nuclear coordinate **R**_*i*_ contributing to the photoabsorption within the energy window is selected as an initial condition, supplemented by the electronic state *J* reached by the initial condition within this window: . The nonadiabatic molecular dynamics is then performed from this set of initial conditions, and the observables and properties deduced from the swarm of trajectories can be convoluted to account for the time spread of the laser pulse. This strategy was applied for several molecular applications, giving access to wavelength-dependent properties like quantum yields.^60^ However, the precise definition of the energy window and the convolution function remains somewhat arbitrary in most applications.

**Figure 5:**
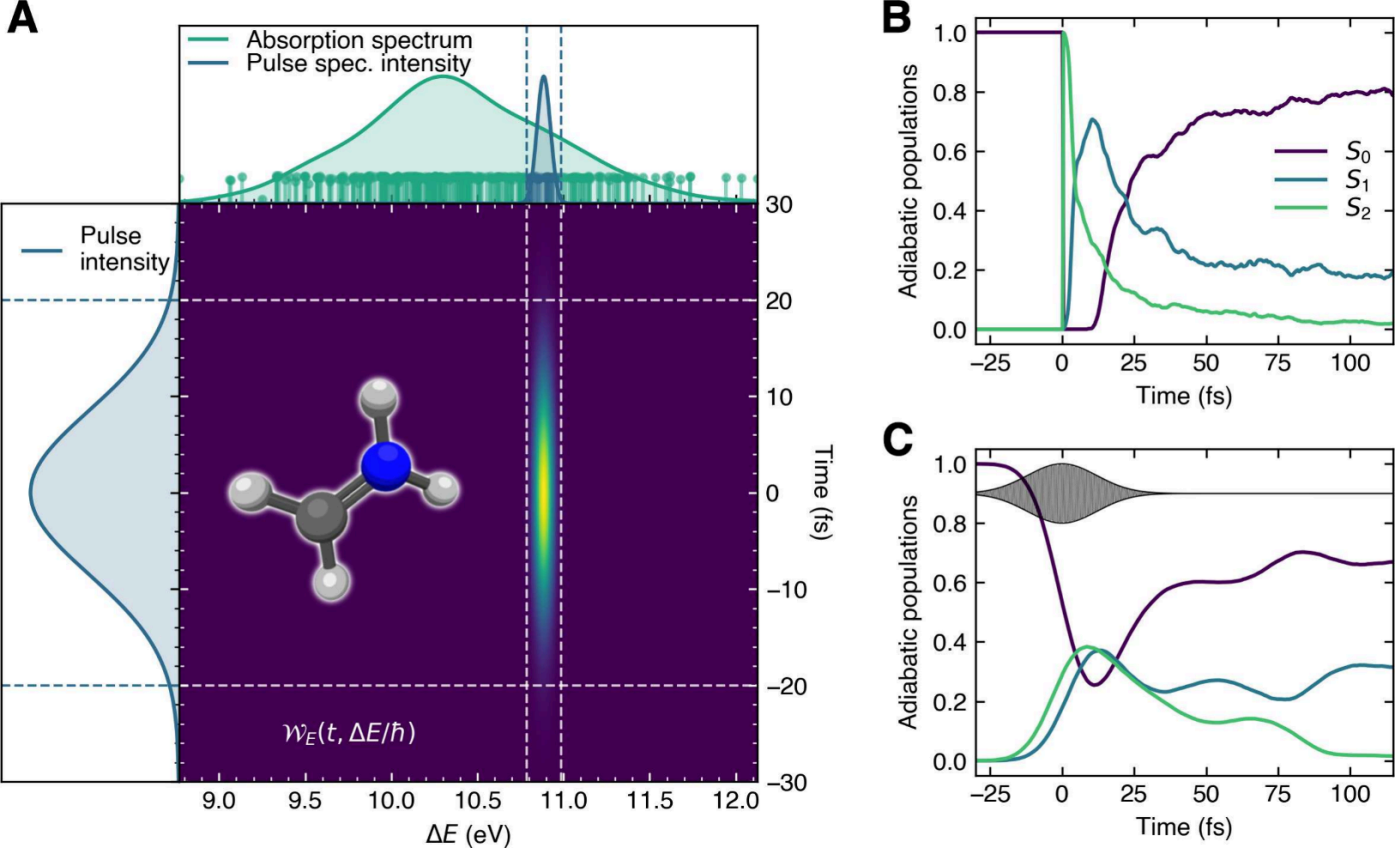
Photodynamics of protonated formaldimine. (A) The Wigner pulse representation  (central panel), the pulse intensity (left panel), and the spectral intensity with the absorption spectrum of protonated formaldimine (upper panel). The excitation energies of the 500 sampled pairs of nuclear positions and momenta are represented as sticks with a height proportional to the square of the transition dipole moment to S_2_. Blue sticks depict the initial conditions selected by PDA. (B) Time trace of the adiabatic electronic populations for TSH with a sudden excitation. (C) Time trace of the adiabatic electronic populations for TSH with PDA. Reproduced from ref (4). Available under a CC-BY license. Copyright 2024 J. Janoš, P. Slavíček, B. F. E. Curchod.

We recently devised a physically based strategy to include the effect of a laser pulse implicitly in the initial conditions for trajectory-based nonadiabatic molecular dynamics, i.e., . This strategy invokes the concept of a *promoted nuclear density*, a virtual quantity that represents the nuclear density promoted to the excited state *J* at time *t*′ which, if propagated in the excited electronic state from all times *t*′ to *t*, would reconstruct the correct excited-state nuclear density at time *t*. Analyzing this promoted nuclear density in the context of trajectory-based methods allows us to include the effect of the laser pulse by correcting the ground-state probability density by the square of the transition dipole moment and the Wigner representation of the pulse (see an example in Figure 5A). These two elements act as a natural filter for the initial conditions, resulting in a time distribution of the nuclear positions and momenta based on the coupling of the laser pulse with the transition dipole moments. Importantly, the so-called promoted density approach (PDA) uses only quantities that would be calculated in a typical sampling of initial conditions within the sudden-excitation approximation and therefore includes the photoexcitation process *at no additional costs*. Figure 5 provides an example of combining PDA with TSH for the protonated formaldimine. The Wigner transform of the pulse applied (Figure 5A) shows its spread in energy and time and can be used to filter initial conditions (here sampled with QT-BOMD, with distributions in energy space depicted on the upper panel). Initiating a TSH dynamics of protonated formaldimine from the second excited electronic state (S_2_) using initial conditions within the sudden approximation (Figure 5B) or the PDA (Figure 5C) leads to dramatically different dynamics for the electronic population, highlighting the importance that the laser pulse may have on the photoexcitation process and the creation of a specific nuclear wavepacket in the excited electronic state(s). For a detailed derivation of PDA and a code to perform the laser-pulse sampling, the reader is referred to ref (4) and its Supporting Information.

Furthermore, an analysis of the promoted nuclear density also provides a justification (and refinement) for the windowing strategy and convolution described above. From the set of sampled nuclear positions and momenta, one should only retain those that lie within the pulse spectral intensity *S*(ω) and apply a weight based on the transition dipole moment and *S*(ω). The resulting observables calculated from the swarm of trajectories initiated from these initial conditions should then be convoluted with pulse intensity *I*(*t*). The precise equations for this strategy coined PDAW (promoted density approach for windowing) are provided in ref (4).

PDA and PDAW offer a simple strategy to move beyond the sudden approximation when describing the photoexcitation resulting from a laser pulse. These techniques are, however, justified only for laser pulses with a duration within 100 fs or under, which is adequate to simulate the outcome of ultrafast spectroscopic experiments but might not be ideal for photoexcitation triggered by continuous lasers, for example.

### 3.2 Photoexcitation with a Continuous-Wave Laser

As stated earlier, short laser pulses are not always used in photochemical experiments. Instead, photochemical reactions are often initiated by radiation at a well-defined wavelength, for example with nanosecond pulses which can be treated almost as continuous-wave (CW) radiation from a theoretical perspective.^1^ In such a scenario, the radiation can form a molecule in one (or a few) specific vibrational eigenstate in the excited electronic state, a sort of state-specific photoexcitation. Another common scenario of photoexcitation departing from short laser pulses is when the molecule is exposed to thermal radiation, such as sunlight.^61^ In these cases, the photodynamics can differ significantly from those observed when the reaction is initiated by a short laser pulse generating a nuclear wavepacket in the excited electronic state(s). Given the strength of trajectory-based nonadiabatic dynamics to describe the rather classical evolution of nuclear wavepackets, one may legitimately ask whether photoexcitation leading to the formation of nearly stationary states (more quantum in nature) can still be mimicked by using (classical) trajectories.

While still an active topic of research, the photochemistry triggered by a CW laser can, in some cases, still be approximated by trajectory-based nonadiabatic molecular dynamics.^1^ A CW laser is essentially a specific example of a laser pulse scenario, and one can generate an excited-state distribution by applying energy filtering based on the resonance condition for a ground-state distribution, which can be sampled using techniques like the QT-BOMD method. However, this approach may become computationally expensive, especially for excitations at the tails of the distribution of the large ground-state sampling required to describe the tails of the distribution sufficiently well. An alternative, considered in ref (1), is to perform ground-state BOMD simulations with a constraint to enforce a given resonance condition (between the desired CW laser frequency and the energy gap between the ground and a given excited electronic state).

Possible future developments in describing state-specific photoexcitation could involve the sampling of a given eigenstate of an excited electronic state using some of the methodologies described in Section 2. Trajectory-based nonadiabatic dynamics could then be launched from these initial conditions, possibly over long time scales (keeping in mind the associated challenge of ZPE leakage discussed above and in refs (42 and 62)). Another possible development would be a more general extension of the PDA to CW pulses, invoking the conceptual idea that a CW pulse is equivalent to a pulsed short laser with a high repetition rate.^63^

## 4 Summary and Outlook

Trajectory-based methods for excited-state and nonadiabatic dynamics are widely used to simulate the photochemistry of molecular systems due to their ease of implementation and conceptual simplicity. While numerous techniques have been proposed to perform nonadiabatic molecular dynamics, our experience shows that the variations in algorithms for nonadiabatic dynamics will typically have a smaller impact on the results of the simulation than an adequate selection of initial conditions. In this Account, we wished to stress that “initial conditions” mean more than just a set of nuclear positions **R**_*i*_ and momenta **P**_*i*_ but should also account for the spectral and temporal properties of the laser pulse, all contained in the descriptor .

For the sampling of nuclear position–momentum pairs, we strongly recommend a careful assessment of the harmonic approximation underlying the harmonic Wigner distribution. As a practical and more robust alternative, we advocate using QT-BOMD, ideally benchmarked against PIMD simulations. While QT-BOMD carries a higher computational cost compared to the harmonic Wigner sampling, the additional benefits are substantial. QT-BOMD performs well for harmonic systems and, importantly, can also handle molecules with multiple minima and moderate anharmonicity. Moreover, QT-BOMD can be used for molecules, molecular clusters, liquids, and solids alike,^2,38,46−48,64,65^ avoiding the combination of incoherent approaches to treat the chromophore and its environment, and using PIMD as a smoke detector for when the method might fail.

The excitation process should also be accounted for in the preparation of the initial conditions. For laser pulses, one can apply energy windowing to the sampled nuclear position–momentum pairs, followed by a time convolution of the observables and properties calculated with nonadiabatic molecular dynamics. The recently proposed PDA strategy offers a more rigorous and systematic approach to incorporate the effect of a laser pulse under the initial conditions and comes at no additional costs.

Finally, we emphasize that even the most advanced protocol for nonadiabatic molecular dynamics cannot compensate for an inaccurate description of the underlying electronic-structure properties (electronic energies, nonadiabatic couplings, etc.). Employing a proper method for the electronic structure remains essential to obtaining reliable results; however, this is a story for a different Account.

## Footnotes

a More precisely, the initial nuclear wave function in the excited electronic state is the ground-state nuclear wave function multiplied by the transition dipole moment between the ground and targeted excited electronic state.

b In addition, it is worth noting that the sudden excitation makes full sense only when considering a system with two electronic states (ground and excited states), as a δ laser pulse is infinitely broad in energy meaning that it would formally excite the molecule in a coherent superposition of all electronic states.

c We keep the notation **R** for the nuclear coordinates, independent of whether it indicates Cartesian or internal coordinates.
